# Iron-containing cookware for the reduction of iron deficiency anemia among children and females of reproductive age in low- and middle-income countries: A systematic review

**DOI:** 10.1371/journal.pone.0221094

**Published:** 2019-09-03

**Authors:** Clark Alves, Ahlam Saleh, Halimatou Alaofè

**Affiliations:** 1 Abrazo Central Campus Family Medicine Residency Program, Phoenix, Arizona, United States of America; 2 Office of Global and Border Health, University of Arizona College of Medicine, Tucson, Arizona, United States of America; 3 Arizona Health Sciences Library, University of Arizona, Tucson, Arizona, United States of America; 4 Department of Health Promotion Sciences, University of Arizona Mel and Enid Zuckerman College of Public Health, Tucson, Arizona, United States of America; University of Virginia, UNITED STATES

## Abstract

**Background & objective:**

There is limited evidence regarding the efficacy of iron-containing pots and ingots in reducing iron deficiency (ID) and iron deficiency anemia (IDA) in low- and middle-income countries (LMICs). The objective of this systematic review is to summarize the evidence regarding the effect of iron-containing cookware on ID and IDA among children and females of reproductive age (FRA) in LMICs.

**Methods:**

Searches were last conducted in May 2019 in PubMed, Embase, Cochrane Library, Web of Science, Scopus, CAB Abstracts, POPLINE, LILACS, ProQuest Dissertations & Theses Global, WHO ICTRP and ClinicalTrials.gov. Hand searching was also conducted. Selection criteria included randomized-controlled trials (RCTs), quasi-experimental studies and observational studies with control groups that studied the effect of iron-containing cookware in children (4 months-11 years) and females of reproductive age (12–51 years).

**Results:**

Eleven studies were eligible for inclusion in the review. Statistically significant increases in hemoglobin and/or iron indices (p < 0.05) were observed in 50% (4/8) of studies on pots (relative change/mean difference in Hb: -0.4–1.20 g/dL), and 33.3% (1/3) of studies on ingots (relative change/mean difference in Hb: 0.32–1.18 g/dL). Positive outcomes (p < 0.05) were observed among children in 50% (4/8) of studies and among FRA in 28.6% (2/7) of studies. Compliance ranged from 26.7–71.4% daily use of pots to 90–93.9% daily use of ingots.

**Conclusions:**

There are indications that, with reasonable compliance, iron-containing cookware could serve as a means of reducing IDA, especially among children. The potential advantages of iron-containing cookware include relative cost-effectiveness and complementary combination with other interventions. However, further research is needed regarding both the efficacy and safety of this intervention.

## Introduction

The reduction of iron deficiency (ID) and iron deficiency anemia (IDA) remains a challenge worldwide, particularly in low- and middle-income countries (LMICs). One billion people worldwide suffer from IDA and a further billion from ID without anemia, with a high proportion of these conditions among children and females of childbearing age [1, 2]. Without dietary fortification, ID is estimated to affect 40% of preschool children, 30% of women of reproductive age and 38% of pregnant women globally [1]. IDA results in poorer educational achievement, impaired cognition, increased mortality and overall morbidity in children, reduced work capacity in adults and poor pregnancy outcomes [1, 3, 4]. In LMICs, the etiology of anemia is multifactorial. Although the main cause is thought to be low dietary iron content and bioavailability, anemia can also result from infections, such as malaria [2], helminthiasis or schistosomiasis [5], chronic inflammatory disorders [1, 3] and nutritional deficiencies of folate, vitamin B12 and vitamin A [1, 2, 6].

Proposed strategies for combating ID and IDA include iron supplementation, artificial iron fortification, biofortification, dietary modification, nutrition education and antiparasitic treatment. Iron supplementation is the most widely implemented approach; however, it has failed as a sole intervention due to challenges regarding cost, distribution, acceptability, political will and sustainability [4, 7, 8]. In addition, iron supplementation can produce undesirable side effects such as abdominal pain, nausea, vomiting and constipation [2]. Artificial iron fortification of staple foods is considered to be the most cost-effective intervention; however, fortification has suffered from the same disadvantages as iron supplementation [4]. Strategic plant breeding and modification of agricultural practices (i.e. biofortification) have emerged as another promising approach to improving iron status among male and female adolescents and adults. However, the ultimate effect on functional outcomes (e.g. hemoglobin levels) remains to be determined, particularly in other vulnerable populations such as children [9].

Dietary modification, which can help alleviate other nutritional deficiencies simultaneously, has never been considered a stand-alone approach due to the higher anticipated costs and limited availability [2, 4, 10]. Indeed, heme iron-rich foods, such as meat, and fruits containing ascorbic acid (which enhances iron absorption) are not always accessible or affordable to many families in LMICs, while cereals, coffee and tea containing phytates, polyphenols and tannins (which all inhibit iron absorption) are often dietary staples [2, 8]. Treatment of malaria, intestinal helminthiasis and/or schistosomiasis is also an effective strategy for reducing IDA [4]. However, since the etiology of IDA is multifactorial, anti-parasitic treatment must be combined with other interventions in most settings [3].

Near the turn of the 21^st^ century, researchers developed an interest in using iron cooking pots as a low-cost, sustainable intervention to reduce IDA by delivering bioavailable iron to food during preparation [11–14]. However, in a systematic review of three eligible randomized-controlled trials (RCTs), Geerligs et al. [15] found that both efficacy and acceptability depended on the size of the pot, age of the intended beneficiaries, whether the pot was used as a replacement or supplemental pot, socio-cultural familiarity with iron pots, and the prevalence of malaria and hookworm infection. In addition, iron pots have been criticized for being difficult to clean, expensive, heavy and unwieldy, exhibiting limited durability with a tendency to rust, and producing iron that is not highly bioavailable [16–19]. In light of the limitations of iron pots, Charles et al. [19] studied an intervention in which food was prepared with an iron fish-shaped figurine (i.e. an ingot). In this RCT, blood iron levels improved among participants, suggesting that the fish-shaped iron ingot had potential for reducing ID. However, further research was required to assess the quantity and bioavailability of iron leached from the ingot. Multiple improvements were later made to the fish-shaped ingot, which led Armstrong et al. to found a for-profit social enterprise in 2012, Lucky Iron Fish Inc., to bring the iron ingot to scale in LMICs [8, 20].

Since the review by Geerligs et al. [15] no updated systematic review has been conducted on the efficacy and acceptability of iron pots. This is despite the fact that several studies have been conducted using pots made of iron alloys, such as blue steel and stainless steel, which are lighter than iron pots and less likely to rust [18]. In addition, no systematic review has been conducted on the efficacy and acceptability of iron ingots in reducing IDA. Therefore, the primary objective of this review is to examine the efficacy of iron-containing cookware (pots and ingots) in reducing ID and IDA among children and females of reproductive age in LMICs. The secondary objectives of this review include examination of iron content and bioavailability in food prepared with iron-containing cookware, as well as assessment of the acceptability, adverse effects and relative cost-effectiveness of iron-containing cookware in reducing IDA. Despite the availability of multiple approaches for addressing IDA, the condition still affects one-fifth of the world’s population, resulting in detrimental effects on health and economic development [2]. Therefore, the public health implications of using iron-containing cookware as a potential tool for reducing IDA cannot be overstated.

## Methods

### Protocol

An a priori protocol was developed, which outlined the objectives and methods of the review. The original protocol was not pre-registered in an official registry, but was provided to PLOS One at the time of submission of this article. The 2009 PRISMA checklist was also completed and included with the submission [21]

### Inclusion criteria

- **Types of studies:** Randomized-controlled trials, quasi-experimental studies and observational studies with a control group.
- **Types of interventions**: Interventions that utilized iron-containing pots, pans and utensils (e.g. iron, blue steel and stainless steel) or ingots (e.g. the Lucky Iron Fish) to reduce iron deficiency (ID) and iron deficiency anemia (IDA) [22–24].
- **Type of comparison**: No intervention or another type of intervention.
- **Target population**: Children four months to 11 years and females of childbearing age (12–51 years). Four months of age was used as a cut-off, since this is the minimum age below which infants are usually exclusively breastfed and thus do not consume significant quantities of complementary foods that would be cooked with iron-containing cookware [25]. The age range of 12–51 years-old was chosen for FRA, since menarche has been noted to begin as early as 11.7 years and menopause as late as 50.7 years in low and middle-income countries [26, 27].
- **Outcomes**: Primary outcomes of interest were hemoglobin concentration (Hb) and iron status. Selected measures of iron status included serum ferritin (SF), serum iron (SFe), total iron binding capacity (TIBC), soluble transferrin receptor concentration (sTFR), serum transferrin (ST) and transferrin saturation (TS). Secondary outcomes of interest included iron content and bioavailability of food, compliance (percentage of participants using iron-containing cookware daily), adverse effects and cost-effectiveness.
- **Setting**: Studies conducted in low and middle-income countries (LMICs). A country’s designation as low- or middle-income was guided by the World Bank’s 2016 Classification of the World Economy criteria [28].

### Exclusion criteria

Studies were excluded if: (1) they were conducted solely on children < 4 months, women > 51 years or males > 11 years, (2) multiple interventions made it impossible to identify the specific effects of iron-containing cookware on the reduction of ID and IDA, (3) they did not include a control group, (4) they did not report on outcomes related to ID or IDA or (5) they were conducted in a high-income country.

### Search strategy

The following resources were searched from inception to May 17, 2019: PubMed (1946-May, 2019), Embase (1947-May, 2019), Wiley Cochrane Library (CDSR, DARE, CENTRAL), Clarivate Analytics Web of Science [Science Citation Index Expanded (1900-May, 2019)] and Conference Proceedings Citation Index-Science (1990-May, 2019), Elsevier Scopus, Ovid CAB Abstracts (1910-May, 2019), POPLINE (1970- May 2019), LILACS, ProQuest Dissertations & Theses Global, WHO ICTRP and ClinicalTrials.gov. The websites of WHO, UNICEF, the United Nations High Commissioner for Refugees (UNHCR) and Lucky Iron Fish, Inc. were also hand-searched. The search strategy was formulated in PubMed and adapted for other databases. The following PubMed search includes the terms and concepts used: ("Anemia"[mesh] OR "Iron, Dietary"[mesh] OR "Iron"[mesh] OR anemia [tiab] OR anemic [tiab] OR anaemia[tiab] OR anaemic [tiab] OR iron* [tiab] OR IDA [tiab]) AND ("Cooking and Eating Utensils"[mesh] OR "Cooking/methods"[mesh] OR "Cooking/instrumentation"[mesh] OR ingot [tiab] OR ingots [tiab] OR "cooking pot" [tiab] OR "cooking pots" [tiab] OR cookware [tiab] OR "cooking pan" [tiab] OR “cooking pans” [tiab] OR "iron pan" [tiab] OR utensil* [tiab] OR "iron pot" [tiab] OR "iron pots" [tiab] OR "iron fish"). The search was not restricted by language or publication date.

In addition, relevant studies cited by identified articles were reviewed. Drs. Stanley Zlotkin and Peter Berti, of the former Appropriate Solutions for Anemia Control project, as well as Dr. Gavin Armstrong, founder and CEO of the Lucky Iron Fish, Inc., were contacted via email to inquire about published studies that may have been overlooked. Drs. Anuradha Khadilkar and Veena Ekbote were contacted for more information and potentially missing data regarding a study published by Kulkarni et al. [29].

### Study selection

Two reviewers (CA and HA) independently screened titles and abstracts of identified studies to include studies based on general relevance to the topic of interest. Second, both reviewers screened the titles and abstracts of studies included in step one to include studies based on reporting of the primary outcomes of interest (i.e. hemoglobin and/or iron status). In this second step, full texts were also screened when reporting on hemoglobin and iron status was unclear. Finally, both reviewers reviewed the full-texts of studies included in step two to assess eligibility based on all population, intervention, comparison and setting (PICOS) criteria. When there was disagreement regarding eligibility, a third team member (AS) was asked to arbitrate.

### Data extraction

Two reviewers (CA and HA) independently extracted data from each eligible study using a Cochrane Collaboration standard data extraction form [30]. Differences were resolved by discussion between reviewers. The following information was extracted from each study: (1) study features (including objective, setting, study design and length of follow-up), (2) number of study participants, (3) type of intervention (including the type of metal from which pots were manufactured), (4) characteristics of the target population (including age and gender), (5) exclusion criteria employed by the study, (6) outcomes of interest and (7) general approach to statistical analysis (i.e. intention-to-treat vs. per-protocol analysis). Studies were also assessed for their consideration of potential effect modifiers for the effect of iron cookware on iron status and anemia: malaria, helminthiasis, schistosomiasis, blood transfusion, iron supplementation and inflammation. The volume (i.e. size) of intervention and control pots was also assessed as a potential effect modifier, as it was found to be related to compliance by Geerligs et al. [15]. In addition, studies were assessed for reporting on the quality of water used for food preparation and the prevalence of genetic hemoglobinopathies. As noted by Charles et al. 2011 [19], water contamination with arsenic and manganese may decrease the bioavailability of iron leached from iron cookware. As noted by Rappaport et al. 2017, individuals with genetic hemoglobinopathies are known to suffer from iron deficiency and anemia, despite adequate iron intake and bioavailability [24, 31]. Since this review involved study-level data only, it was not submitted for IRB review.

### Methodological quality assessment

Two reviewers (CA and HA) independently performed the methodological quality assessment of included studies using the risk of bias criteria for randomized-controlled trials, non-randomized controlled trials and controlled before-and-after studies developed by the Cochrane Effective Practice and Organization of Care group [32]. These nine criteria include random sequence generation, allocation concealment, similarity of baseline outcome measurements, similarity of other baseline characteristics, complete outcome data, adequate blinding, protection against contamination, non-selective outcome reporting and no other risk of bias. In addition, this approach was combined with that employed by Verhagen et al. [33]. That is, each criterion was scored, receiving 1 point if the criterion was fully met, 0.5 points if the criterion was partially met and 0 points if the criterion was poorly met.

‘Other bias’ was defined as bias that could result from factors not captured by the other eight criteria. A score of 1 was assigned if there was little to no perceived risk of other bias, a score of 0.5 was assigned if there was only one potential source of other bias (i.e. moderate risk of other bias) and 0 was assigned if there were at least two additional sources of potential bias (i.e. high risk of other bias). Utilizing per-protocol analysis (instead of intention to treat) was considered to represent moderate risk of other bias. As described by Ranganathan et al. (2016), intention-to-treat analysis is important for intervention-based RCTs, since it minimizes the risk of potential confounding variables achieved with randomization, ensures adequate simple size and reduces risk of other bias [34]. The criterion was scored as ‘NS’ if the information was unclear or not specified. The total score for each study was calculated by adding these subscores. Also in accordance with Verhagen et al. [33], methodological quality was considered ‘high’ from 8 to 9, ‘moderate’ from 4 to 7 and ‘low’ from 1 to 3. Loss to follow-up ≤ 20% was considered acceptable, since loss-to-follow-up greater than 20% poses serious risks to validity [33, 35]. Adequate follow-up was captured by the criterion ‘complete outcome data.’

### Data analysis

Due to high heterogeneity among studies, the decision was made to not conduct a meta-analysis. For example, some studies reported p-values for relative changes in Hb concentration in the intervention and control groups, while others reported p-values for the mean difference between the groups at endline. When p-values were reported for relative changes in the intervention vs. control groups, the following equation was used calculate a standardized relative change to better quantify these changes and facilitate comparisons between studies:

Relativechange=changeininterventiongroup–changeincontrolgroup

For example, if Hb increased by 0.5 g/dL in the iron pot group, while decreasing by 0.7 g/dL in the aluminum pot group, the standardized relative change would be 0.5-(-0.7) = 1.2 g/dL. When p-values were reported only for mean differences in Hb at endline, this equation was not used to calculate a standardized relative change. Relative changes were reported preferentially in Table 1, as relative changes account for differences in baseline Hb, whereas mean differences at endline do not. A similar equation was used to calculate standardized relative changes in iron indices when p-values were reported for relative change.

**Table 1:** General characteristics and outcomes of included studies (Green: High quality, Yellow: Moderate quality, Red: Low quality).

Author (year)	Country	Duration	Study design	Participants	Exclusion Criteria	Intervention	Main Findings
Devadas et al. (1973)	India	7 months	Quasi-experimental	140 children in a school lunch program	Not participating in school lunch program	Consumption of food prepared in iron vs. aluminum pots and other controls	Relative change^a^ in Hb in iron vs. aluminum pot groups: +0.64 g/dL, p < 0.01; relative increase of 95 mg of Fe/100 g of food when amaranth prepared in iron vs. aluminum pot (p < 0.01)
Borigato and Martinez (1998)	Brazil	8 months	RCT	63 preterm infants (4 mos. postnatal age)	Severe illness, blood transfusion	Consumption of food prepared in pig iron vs. aluminum pots	Relative change in Hb in iron vs. aluminum pot groups: +1.2 g/dL, p = 0.01; relative change in SFe in iron vs. aluminum pot groups: + 14.4 μmol/L, p = 0.04
Adish et al. (1999)	Ethiopia	12 months	RCT	407 children aged 2–5 y	Severe illness, chronic disorder, physical disability, Hct < 20 or > 34%	Consumption of food prepared in iron vs. aluminium pots	Adjusted mean difference in Hb in iron vs. aluminum pot groups^c^: +1.2 g/dL (p < 0.001); mean difference in SF in iron vs. aluminum groups: +12.7 μg/L (p < 0.001); available Fe 5 times greater after preparation in iron vs. aluminum pot (0.24 vs. 0.05 mg/100 g of food); ^b^equivalent daily use 42%
Geerligs et al. (2003)	Malawi	5 months	RCT	322 participants ≥ 1 y, 128 < 12 y and 194 ≥ 12 y	Hb < 7.0 g/dL, pregnant w/ Hb < 8.0 g/dL, iron supplements, blood transfusion	Consumption of food prepared in cast iron vs. aluminum pots	< 12 years: no significant change or difference in Hb; ≥ 12 years: relative change in Hb in iron vs. aluminum pot groups +0.75 g/dL, p = 0.01; < 12 years: relative change in ZP in iron vs aluminum groups -2.8 μg ZP/g Hb (p < 0.05); ≥ 12 years: no significant change or difference in ZP, daily use: 31.1%
Berti et al. (2004)	Vietnam	5 months	Cluster RCT	65 infants 6–24 mos, 121 girls 11–14 y, and 172 FRA 15–43 y	Household with at least one anemic individual from target groups	Consumption of food prepared in cast iron vs. blue steel pots vs. no intervention	Overall relative change in Hb in consistent users of iron pots vs. control: WRA^d^ +0.3 g/dL, adolescent girls -0.3 g/dL, infants -0.4 g/dL, similar findings for blue steel pots, all p > 0.05; overall relative change in SF in consistent users of iron pots vs. control: WRA +5 μg/L, adolescent girls -8 μg/L, infants +12 μg/L, similar findings for blue steel pots, all p > 0.05; daily use 34% for iron, 38% for blue steel
Sharieff et al. (2008)	Benin	6 months	Cluster RCT	71 children 6–24 mos, 92 adolescent girls 11–15 y, and 131 FRA 15–44 y	Transfusion, iron supplements, plan to emigrate within 6 mos, Hb < 7.0 g/dL	Consumption of food prepared in cast iron vs. blue steel pots vs. iron supplement	Mean difference in Hb in iron pot vs. supplement groups: 0.0 g/dL (p = 0.73), similar findings for blue steel pots; mean difference in SF in iron pot vs. supplement groups: -22 μg/L (p < 0.0001), similar findings for blue steel pots; equivalent daily use 25.7% for both pot types
Talley et al. (2010)	Tanzania	12 months	Controlled before-after with cross-sectional surveys	110 children 6 mos to 5 y and their mothers (18–58 y)	Pregnant, permanent emigration from camp, no residence in camp at baseline	Consumption of food prepared in stainless steel vs. aluminum/ clay pots	Children: no significant differences or changes in Hb; Mothers: mean difference in Hb in stainless steel vs. aluminum/clay pot groups -0.3 g/dL (p = 0.485); Children: mean difference in sTFR in stainless steel vs. aluminum/clay pots groups -0.9 μg/L (p < 0.001); Mothers: mean difference in sTFR in stainless steel vs. aluminum pot groups -1.1 μg/L (p = 0.003); equivalent daily use 26.7%
Arcanjo et al. (2018)	Brazil	4 months	Cluster RCT	175 children 4–5 y	Refusal to participate, taking iron supplements	Consumption of food prepared in iron vs. aluminum pots	Relative change in Hb in iron vs. aluminum pot groups: +0.26 g/dL (p = 0.16); Hb change in anemic children iron pot group: +1.69 g/dL (p < 0.0001); Hb change in anemic children aluminum pot group: +1.10 g/dL (p = 0.02); equivalent daily use 71.4%
Charles et al. (2011)	Cambodia	6 months	RCT	189 pre- and post-menopausal women > 16 years	Hct < 30% at baseline, CRP ≥ 6.0 mg/L at endline	Iron ingot vs. iron ingot + educational follow-up vs. no intervention	β for changes/differences in Hb in ingot w/ follow-up vs. control groups: +0.18 (p = 0.51); β for changes/differences in SFe in ingot w/ follow-up vs. control groups: -4.5 (p = 0.23)
Charles et al. (2015)	Cambodia	12 months	RCT	310 pre- and post-menopausal women > 16 years	Hb < 7.0 g/dL, plan to migrate before end of trial, pregnant, iron supplements in past 3 mos, Hb < 12 g/dL w/ CRP > 10 mg/L	Iron ingot vs. iron ingot + educational follow-up vs. no intervention	Mean difference in Hb in combined iron ingot groups vs. control: +1.18 g/dL (p < 0.0001); mean difference in SF in combined ingot groups vs. control: +31.0 ng/mL (p < 0.001); daily use 93.9%
Rappaport et al. (2017)	Cambodia	12 months	RCT	327 FRA 18–49 years	Hb < 8.0 or ≥ 12.0 g/dL, not female head of household, ill health, pregnant, medications or iron supplements, participating in another nutrition intervention, plan to migrate	Lucky Iron Fish vs. iron supplement vs. no intervention	Mean difference in imputed Hb in ingot vs. no intervention groups: +0.32 g/dL (p = 0.850); imputed mean difference in SF in ingot vs. no intervention groups: +0.96 μg/L (p = 0.781); mean difference in imputed sTFR in ingot vs. no intervention groups: +1.00 mg/L (p = 0.997); daily use 90%

FRA, females of reproductive age; TS, transferrin saturation; SFe, serum iron; FEP, free erythrocyte protoporphyrin concentration; SF, serum ferritin concentration; ZP, zinc protoporphyrin level; sTFR, serum transferrin receptor concentration; β, coefficient of effect after adjustment with multiple linear regression; IDA, iron deficiency anemia

^a^Relative change in Hb = change in intervention group–change in control group

^b^Equivalent daily use = (days per week of use/7 days per week) x % of participants achieving reported minimum days of use

^c^All mean differences correspond to differences at study endline

^d^WRA, women of reproductive age 15–43 yo, adolescent girls in this study were 11–14 yo, infants were 6–24 mos

When studies reported compliance as the percentage of participants using cookware at a frequency less than daily, this percentage was converted to correspond to daily use (i.e. equivalent daily use).

Equivalentdailyuse=(daysofuseperweek/7daysperweek)xpercentageofparticipantsachievingminimumreporteddaysofuse

For example, if cookware was used at least three times per week by 60% of participants, the equivalent percentage of daily use would be (3 days/7 days) x 60% = 25.7%. This also allowed for more direct comparison between studies. Overall, analysis of the 11 eligible studies was conducted by summarizing, comparing and contrasting the extracted data.

## Results

**Fig 1:**
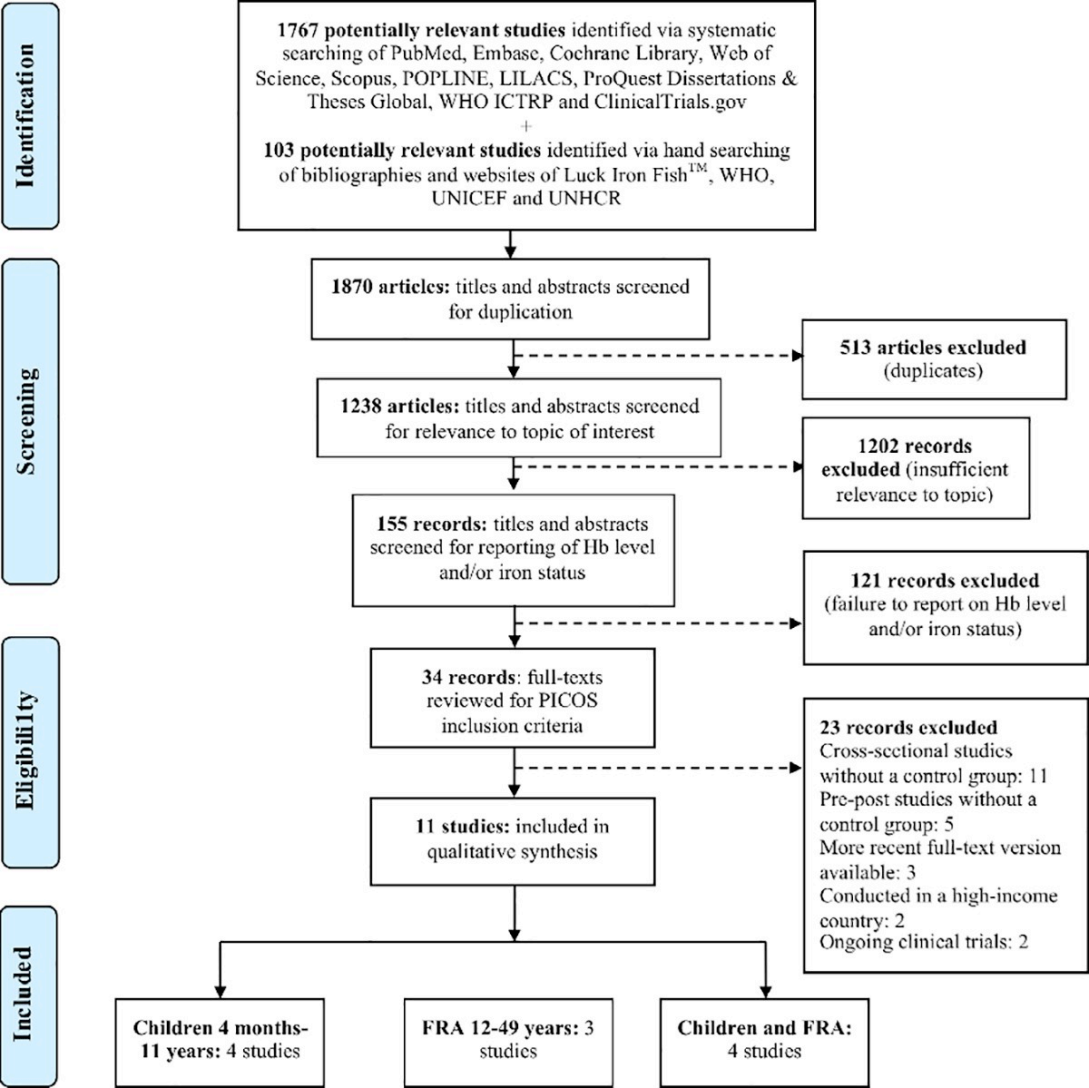
Flow chart of study selection process. FRA: females of reproductive age.

Initially, 1870 records were identified. Following review of titles and abstracts for relevance to the topic of interest, 155 records were considered potentially eligible for inclusion. Further review of the titles and abstracts of these records for reporting of Hb concentration and/or iron status resulted in 34 potentially eligible studies. Sixteen studies lacked a control group, three studies had a more recent full-text version available, two studies were conducted in a high-income country and two studies were ongoing clinical trials. Of the resulting 27 studies, 16 were excluded for not meeting the PICOS inclusion criteria. The resulting 11 studies were included in this review (Fig 1). The main findings are divided into five subsections: (1) sample characteristics, (2) intervention characteristics, (3) intervention efficacy, (4) potential effect modifiers and (5) quality assessment.

### Sample characteristics

As shown in Table 1, the included studies were conducted in Asia (Cambodia, India and Vietnam), Africa (Benin, Ethiopia, Malawi and Tanzania) and Latin America (Brazil). They were published between 1973 and 2018 and consisted of nine randomized-controlled trials, one controlled before-and-after study with cross-sectional surveys and one quasi-experimental trial. All studies were published in English. Interventions in the studies took place in rural, semi-urban and urban settings. Participants were identified in multiple venues, including schools, hospitals, refugee camps and communities, and through a variety of methods, including random selection as well as follow-up of cross-sectional surveys and other studies of anemia prevalence and etiology. In Table 1, high quality studies are coded in green, moderate quality studies in yellow and low quality studies in red.

Across the 11 included studies, there was significant heterogeneity in sample characteristics, including size, age and exclusion criteria. In addition, there was marked variation in follow-up length and the format for outcome reporting. Samples sizes ranged from 63–407 for children, 64–327 for FRA, ages from 4 months-12 years for children, 12–49 years for FRA and follow-up length from 4–12 months. Four studies involved post-menopausal women up to the age of at least 58 years. Also, two studies were conducted in an urban area [11, 17], eight studies were conduct in a semi-urban or rural area [12, 13, 19, 22, 24, 36–38] and one study was conducted in an area of unknown urban/rural status [39]. Four studies reported mean difference in Hb at endline as the main outcome of interest [12, 17, 22, 24], three studies reported relative change in Hb only [36, 38, 39], two studies reported both mean difference at endline and relative change [11, 13], one study reported a β-coefficient from multiple regression [19] and one study reported both the mean difference at endline and a β-coefficient [37].

### Intervention characteristics

Identified studies could be classified across two different axes: (a) those involving iron-containing pots vs. iron ingots and (b) those involving primarily children vs. FRA. Specifically, five studies focused on consumption of food prepared in iron pots, two studies focused on consumption of food prepared in iron and blue steel pots and one study employed stainless steel pots only. Control groups employed in these studies included groups using aluminum pots, iron supplementation tablets or Micronutrient Sprinkles, as well as groups receiving no intervention. In Devadas et al. [39], utensils were understood to refer to receptacles used for cooking food (i.e. pots), rather than to forks, spoons or knives. None of the included studies used iron pans. Capacity of the iron-containing pots ranged from 2 to 20 liters. Three studies focused on use of an iron ingot, later trademarked as the Lucky Iron Fish, with and without follow-up education to reinforce compliance. Control groups employed in these studies included iron supplementation tablets and no intervention. Four of the eleven included studies targeted only children, three studies targeted only FRA and four studies targeted both children and FRA (Table 1).

### Intervention efficacy

#### Iron-containing pots for reduction of IDA

There was marked variation in the effect of iron-containing pots on anemia and iron status. Qualitatively, much of this variation appeared to be explained by differences in the target population studied and intervention compliance. For example, Devadas et al., Borigato and Martinez, Adish et al. and Arcanjo et al. included only children in their studies (Table 1). In Devadas et al., Hb increased 1.86 g/dL when iron-rich amaranth was prepared in iron pots vs. an increase of 1.22 g/dL when amaranth was prepared in aluminum pots (relative change: +0.64 g/dL, p < 0.01) [39]. In Borigato and Martinez, Hb increased 0.5 g/dL when consumed food was prepared in iron pots, but decreased 0.7 g/dL when food was prepared in aluminum pots (relative change: +1.2 g/dL, p = 0.02) [11]. In Adish et al., Hb increased 1.7 g/dL in the iron pot group, but only 0.4 g/dL in the aluminum pot group (adjusted mean difference: +1.2 g/dL, p < 0.001) [12]. In Arcanjo et al., the overall improvement in Hb in the iron pot group was not statistically significant (+0.04 g/dL, p = 0.78). There was an overall net decrease in Hb in the aluminum pot group that was also not statically significant (-0.22 g/dL, p = 0.07). However, among children who were anemic at baseline, there was a statistically significant increase in Hb in both groups, although the increase was greater in the iron pot group (+1.69 g/dL, p < 0.0001) compared to the aluminum pot group (+1.10 g/dL, p = 0.02). The overall relative change in Hb, regardless of anemic status, was +0.26 g/dL in the iron vs. aluminum pot groups (p = 0.16) [38]. Of these three studies, Adish et al. and Arcanjo et al. assessed daily compliance, found to be 42% and 71.4%, respectively [12, 38].

Sharieff et al. studied both children and females of reproductive age. In this study, Hb increased only 0.3 g/dL in the iron pot group vs. 0.5 g/dL in the iron supplement group (mean difference: 0.0 g/dL at 6 mos., p = 0.73), with similar findings for blue steel pots. However, daily compliance was only 25.7%. [17]. Similarly, Talley et al. studied children and their mothers. Among children in this study, there were no significant changes or differences in Hb. Among mothers, Hb decreased 0.6 g/dL in the stainless steel group, while increasing 0.5 g/dL in the aluminum and clay pot group (mean difference at 12 mos: -0.3 g/dL, p = 0.485). Daily compliance was similar to that found in the study by Sharieff et al. (26.7%) [22]. Variation in the effect of iron-containing pots on iron status showed similar patterns among these five studies.

Geerligs et al. conducted an intervention that lies midway on the spectrum of compliance. This study involved both children and adolescents/adults, and reported a daily compliance of 31.1%. Among adolescents and adults, Hb increased 0.53 g/dL in the iron pot group, while decreasing 0.22 g/dL in the aluminum pot group (relative change: +0.75 g/dL, p = 0.01). Among children < 12 years, there were no significant changes or differences in Hb. However, among children < 12 years, zinc protoporphyrin (as an inverse measure of iron status) exhibited a relative decrease of -2.8 μg ZP/g Hb in the iron pot group compared to the aluminum pot group (p < 0.05). Among adolescents and adults, there were no significant differences in zinc protoporphyin at endline [13]. The intervention by Berti et al. involved infants and females of reproductive age, compliance was similar to that reported by Geerligs et al. (34–38%) and findings were similar to those reported by Sharieff et al. and Talley et al. [36]. However, this study may represent an outlier, since it was the only study in this review considered to be low quality.

Iron content and availability of food increased with the use of iron-containing pots in both studies in which these outcomes were assessed [12, 39]. Overall, statistically significant increases in Hb were observed among children in 37.5% (3/8) of studies (relative change/mean difference in Hb at endline: 0.64–1.2 g/dL) (Table 2). In one additional study, a statistically significant increase in iron status was observed among children [13]. Statistically significant changes in Hb were observed among females of reproductive age in only 25% (1/4) of relevant studies (relative change/mean difference in Hb at endline: 0.75 g/dL) (Table 2). Surprisingly, in this study, there was no statistically significant increase in iron status among FRA [13]. The overall range of relative change/mean difference in Hb at endline for all studies was -0.4–1.2 g/dL (Table 1).

**Table 2:** Number and percentage of studies demonstrating statistically significant increases/differences in Hb and Fe status among children vs. FRA.

**Iron Pots**	*Children* *#(%)*	*FRA* *#(%)*	*Total* *#(%)*
*Increase in Hb*	3 (37.5%)	1 (25%)	4 (50%
*Increase in Fe Status*	4 (50%)	0 (0%)	4 (50%)
*Total*	8	4	8
**Iron Ingots**	*Children* *#(%)*	*FRA* *#(%)*	*Total* *#(%)*
*Increase in Hb*	NA	1 (33.3%)	1 (33.3%)
*Increase in Fe Status*	NA	1 (33.3%)	1 (33.3%)
*Total*	NA	3	3

#: Number of studies

%: Percentage of studies

#### Iron ingots for reduction of IDA

There was also significant variation in the effect of iron ingots on Hb and iron status. However, this variation did not appear to be explained by differences in intervention compliance. In addition, comparisons between children and FRA could not be made, as only the latter population group was studied. In 2011, Charles et al. studied pre- and post-menopausal women. At midline, mean Hb and serum iron were higher in the intervention group utilizing an iron ingot and receiving educational follow-up, as compared to the control group. However, at endline, there were no significant differences in Hb or serum iron between the intervention and control groups [19]. In 2015, Charles et al. conducted a similar study of pre- and post-menopausal women. Mean Hb in the combined intervention groups increased 1.3 g/dL compared to an increase of only 0.1 g/dL in the control group (mean difference at 12 mos: 1.18 g/dL, p < 0.0001). A similar difference was observed for serum ferritin at endline. Compared to iron-containing pots, daily compliance with use of iron ingots was remarkably high at 93.9% in this study [37]. In 2017, Rappaport et al. studied only females of reproductive age. Significant loss to follow-up necessitated imputation. However, after both imputation and complete-case analysis, there were no significant changes or differences in Hb or serum ferritin. Compliance was again high at 90.0%. Overall, 1 of 3 studies (33.3%) involving iron ingots demonstrated positive changes in Hb and iron status at endline (Table 2). The overall range of relative change/mean difference in Hb at endline was 0.32–1.18 g/dL (Table 1).

### Effect modifiers

As evident in Table 3, there was variable reporting of effect modifiers, including the prevalence of malaria, intestinal helminthiasis and schistosomiasis, blood transfusion, iron supplementation, pot volume, inflammatory state, water contamination and the prevalence of genetic hemoglobinopathies. Eight (of eleven) studies did not report on malaria endemicity and nine studies did not report on helminthiasis or schistosomiasis prevalence. Although two studies employed receipt of blood transfusion as an exclusion criterion at baseline, only one (of eleven) clearly monitored blood transfusions during follow-up. Similarly, although four studies specified iron supplementation as an exclusion criterion at baseline, only three studies clearly monitored iron supplementation during follow-up. Six (of eight) studies reported on pot volume, yet only three of these studies reported on the volume of both intervention and control pots. Only four studies adjusted outcome data for inflammation and/or excluded participants presumed to be suffering from inflammatory anemia. Only Charles et al. 2011 [19] mentioned the role of water quality in influencing the bioavailability of iron in drinking water. However, the authors did not compare the quality of water used to prepare food in the various arms of the study. Similarly, only Rappaport et al. [24] reported on the prevalence of genetic hemoglobinopathies, with 69% of study participants testing positive for a structural hemoglobin variant, and 59.8% of the remaining participants demonstrating evidence for carriage of traits for alpha and/or beta-thalassemia (low mean corpuscular volume despite adequate stores of iron).

**Table 3:** Description of potential effect modifiers.

Author (year)	Malaria prevalence	Helminthiasis and schistosomiasis prevalence	Hemoglobinopathy prevalence	Blood transfusion given	Use of iron supplements	Pot volume	Adjustment for inflammation
Devadas et al. (1973)	Not reported	Not reported	Not reported	Not reported	Not reported	Not reported	No
Borigato and Martinez (1998)	Not reported	Not reported	Not reported	Exclusion criterion at baseline	Iron supplementation was recommended for all participants from 15 days to 12 months of age. Otherwise, iron-fortified cereals/formulas were not used during the study.	Iron: 2 L Aluminum: Not reported	No
Adish et al. (1999)	Very low	Very low	Not reported	Not reported	Not reported, but mothers in the aluminum pot group received iron supplementation for first 3 months	Iron: 2 L Aluminum: 2 L	No
Geerligs et al. (2003)	45.3% in children < 12 years vs. 17.5% in children ≥ 12 years at endline (p < 0.001)	Not reported	Not reported	Participants excluded if blood transfusion given during follow-up	Participants excluded if iron supplements taken during follow-up	Iron: 10 L Aluminum: 6 L	No
Berti et al. (2004)	Not reported, but no seasonal environmental changes during study	Not reported	Not reported	Not reported	Not reported, but no increased self treatment during study	Not reported	Unclear
Sharieff et al. (2008)	Not reported	Not reported	Not reported	Exclusion criterion at baseline, but unclear if transfusion during study was assessed	Exclusion criterion at baseline, also an intervention arm, unclear if iron supplementation in other arms assessed during study	Iron: 2 L Blue steel: Not reported	Yes
Talley et al. (2010)	No significant difference in infectious illness between groups over time (including malaria)	No significant difference in infectious illness between groups over time (including hookworm and schistosomiasis)	Not reported	Not reported	Iron-fortified corn-soya blend was introduced into the general ration before study initiation. Among mothers, iron supplementation decreased in the intervention camp between baseline and 12 months, but no such trend was seen in the control camp. Among children, iron supplementation decreased equally in both groups during the study.	Stainless steel: 5 L Aluminum and clay: not reported	No
Arcanjo et al. (2018)	Not reported	Not reported	Not reported	Not reported	Exclusion criterion at baseline	Iron: 20 L Aluminum: 20 L	No
Charles et al. (2011)	Not reported	Not reported	Not reported	Not reported	88.4% of participants may have received 1 month of iron supplementation therapy 2 months before recruitment	NA	Yes
Charles et al. (2015)	Not reported	Not reported	Not reported	Not reported	Exclusion criterion at baseline, women who used iron supplements during the trial were removed at endline (n = 0)	NA	Yes
Rappaport et al. (2017)	Not reported	Not reported	93–94% across all 3 groups	Not reported	Exclusion criterion at baseline, intervention arm, unclear if iron supplementation in other arms assessed during study	NA	Yes

### Quality assessment

The methodological quality of the included studies was sufficient, but not optimal. As shown in Table 4, seven (of nine) RCTs reported random sequence generation. Two (of ten) studies reported concealment of allocation. Eight (of ten) studies reported similar baseline outcome measurements. One additional study [22] met this criterion partially, as the baseline Hb of mothers in the intervention camp (Nduta) was 14.5 g/dL, while the baseline Hb in the control camp (Mtendeli) was 13.7 (p < 0.001). In eight studies, other baseline characteristics were similar. Five studies reported complete outcome data. Two studies did not fulfill this criterion due to loss-to-follow-up greater than 20% across all groups. Four studies fulfilled this criterion partially, as they experienced greater than 20% loss-to-follow-up in only one group, or were able to employ compensatory data analysis. In five studies, unintentional crossover between intervention and control groups was minimized or prevented (i.e. protection against contamination). Two studies had no risk of other bias, three studies had moderate risk of other bias and six studies had high risk of other bias. Overall, one study was determined to be of high quality (score 8–9), nine studies of moderate quality (score 4–7) and one study of low quality (1–3) (Table 4).

**Table 4:** Quality assessment of included studies.

Study	Random sequence generation	Allocation concealment	Baseline outcome measurements similar	Baseline characteristics similar	Complete outcome data	Adequate Blinding	Protection against contamination	Nonselective outcome reporting	No risk of other bias	Total Score
Devadas et al. (1973)	0	NS	1	1	1	1	0.5	1	0.5	6.0
Borigato and Martinez (1998)	NS	NS	1	1	1	1	0.5	0.5	0.5	5.5
Adish et al. (1999)	1	1	1	1	1	1	0.5	1	0	7.5
Geerligs et al. (2003)	1	NS	0	1	0	1	1	1	1	6.0
Berti et al. (2004)	1	0	0	NS	0	1	1	0.5	0	3.5
Sharieff et al. (2008)	1	1	1	1	1	1	1	1	1	9.0
Talley et al. (2010)	0	0	0.5	0	0.5	1	1	1	0	4.0
Arcanjo et al. (2018)	1	NS	1	1	1	1	0.5	1	0	6.5
Charles et al. (2011)	1	0	1	1	0.5	1	NS	1	0	5.5
Charles et al. (2015)	1	0	1	1	0.5	1	NS	1	0	5.5
Rappaport et al. (2017)	1	NS	1	0.5	0.5	1	NS	0.5	0.5	5.0

High quality: total score 8–9

Medium quality: total score 4–7

Low quality: total score 1–3

NS = criteria not reported or unclear

## Discussion

The studies included in this review suggest that, with reasonable compliance, iron-containing pots and ingots could be used to reduce iron deficiency anemia, especially among children. However, the ultimate effect of iron-containing cookware on iron deficiency anemia varied significantly. Of the 8 studies involving iron-containing pots, 3 (37.5%) demonstrated statistically significant increases and/or differences in hemoglobin levels when compared to non-iron-containing cookware. In 4 out of 8 studies (50%), there were also statistically significant increases and/or differences in iron status. Of the 3 studies involving iron ingots,1 (33.3%) demonstrated statistically significant increases and/or differences in hemoglobin and iron status vs. no intervention at endline (Charles et al. 2015). Overall, children experienced statistically significant increases and/or differences in Hb and/or iron status in 4 of 8 studies (50%), while FRA exhibited these outcomes in only 2 of 7 studies (28.6%). Further research is needed before more firm conclusions can be made regarding the efficacy of iron cookware for reduction of IDA in LMICs.

### Limitations

Careful analysis of the studies included in this review reveals a number of limitations. First, there was variable presence and reporting of potential effect modifiers, including the prevalence of malaria, intestinal helminthiasis and schistosomiasis, blood transfusion, iron supplementation, pot volume and inflammatory state. However, based on the available data, these potential effect modifiers did not appear to explain the variation in study outcomes. In contrast, as noted by Charles et al. (2011) and Rappaport et al. (2017), respectively, water contamination and the prevalence of genetic hemoglobinopathies may have been more influential [19, 24]. Since contamination of water with arsenic and manganese is common in LMICs [40, 41], water quality may have been an unmeasured effect modifier in many studies in this review. In addition, as the prevalence of genetic hemoglobinopathies varies significantly by geographic region [42], this may have been another important effect modifier.

A second limitation of the studies presented here was limited discussion of factors affecting compliance. In a study of the same population recruited by Geerligs et al. 2003 [13], Geerligs et al. 2002 [16] investigated the factors affecting the acceptability of iron pots. The authors noted that iron pots were not used traditionally in the intervention community, which may have accounted for the greater loss to follow-up in the intervention group in Geerligs et al. 2003 [13]. Besides low cultural acceptability, another disagreeable aspect of the iron pots was their greater weight. A similar study by Tripp et al. [18] preceded and accompanied the study by Talley et al. [22]. In this study, authors investigated the acceptability of cast iron, blue steel and stainless steel pots compared to aluminum pots. Limitations of all pot types, except stainless steel, included heavier weight and rust formation. In addition, even though cast iron and blue steel pots were less expensive and leached greater quantities of iron, stainless steel pots were lighter, less likely to rust and presumably less likely to cause iron overload [18]. As a result, stainless steel pots were selected for use in the study by Talley et al. [22]. However, Tripp et al. [18] also reported on the limitations of the stainless steel pots used in Talley et al. [22], which included difficulty with using and cleaning, as well as the tendency for households subsisting on minimal income to sell the stainless steel pots, while continuing to use existing aluminum and clay pots. As a result, Tripp et al. [18] advised against the use of stainless steel pots in a setting where poverty and the presence of other pots of less market value may lead to selling of supplemental stainless steel pots.

Interestingly, although the study involving iron and blue steel pots by Berti et al. [36] was abandoned at midline due to logistical challenges, the investigators had developed a promising prototype pot with an inner layer of steel and an outer layer of aluminum, which made the pot light and attractive, while still facilitating iron leaching. However, the pot also had a layer of air between the aluminum and steel layers, which delayed heating and decreased the overall popularity of the pot (P. Berti, 2018, personal communication). Most importantly, the metal from which pots were made did not appear to explain the variation in outcomes observed in the studies included in this review.

A third limitation of the studies included in this review was the reporting of incomplete outcome data. For example, in Talley et al. [22], a sample size of 100 for each cross-sectional survey conducted during the study was predicted to yield sufficient power to detect a significant change and/or difference in relevant outcomes. Among children, sample sizes were adequate. However, among mothers, the number of participants for whom data was available with each survey ranged from 64 to 85. In Charles et al. [19], loss-to-follow-up was 36.5% across all groups; however, as the authors note, there were no significant differences in baseline Hb or SFe between participants who did and did not complete the study. In Charles et al. [37], some data was not reported: including baseline Hb and CRP by village (claimed to be significantly different), as well as the p-values for associations between loss-to-follow-up and baseline iron status, and between Hb, SFe and menstrual status (claimed to be non-significant). In Rappaport et al. [24], based on a predicted final sample size of 270 participants, a mean hemoglobin difference of 0.5 g/dL could be detected with 90% power and α = 0.05. Although 327 women entered the study, 240 remained after 12 months (26.6% loss to follow-up). However, imputation was employed, and authors reported that results achieved with imputation did not differ from those acquired with complete-case analysis (although complete-case data were reported in supplemental tables that were not readily apparent in the publication).

A fourth limitation of the studies in this review was the presence of moderate to high risk of other bias. For example, in the studies by Charles et al. 2011 and 2015 [19, 37], the authors do not mention a potential conflict of interest, as the ingot used in these studies was refined and marketed by the for-profit social enterprise *Lucky Iron Fish*, *Inc*. While it is unclear if the authors of these studies intended to ultimately market the ingot worldwide, and the founder and CEO of *Lucky Iron Fish*, *Inc*. (Gavin Armstrong) was not listed as one of the authors in these studies, the company was established in 2012, before publication of the second study by Charles et al. in 2015 [20, 43]. In addition, in Adish et al. [12], rates of diarrhea, acute respiratory illness and fever decreased more in the iron pot group than in the aluminum pot group during the study. This may have led to decreased rates of inflammatory anemia and therefore greater perceived improvement of anemia in the iron pot group. However, this decrease in infectious illness could have also been due to improved immune function due to improved iron status [44]. Serum ferritin was assessed in this study but was not correlated with Hb concentration to distinguish IDA from other forms of anemia. In addition, SF was collected in a sample of 170 children early in the study and later in a different sample of 84 children, thereby introducing potential biases due to utilizing a different sample of participants.

In Berti et al. [36], the authors suggested that because they sought out anemic individuals for their study, the global improvement in Hb may have been due to regression-to-the-mean effect. In addition, although CRP was measured at baseline and midline in this study, it was not apparent if this data was factored into the ultimate analysis of data. In Talley et al. [22], data was gathered through multiple random samples of the study population, each of which would have captured a slightly different set of participants. Additionally, in a departure from previous studies, cutoffs for anemia were adjusted for altitude. In Arcanjo et al. [38], the paradoxical improvement in Hb among anemic children in both the iron and aluminum pot groups, despite a statistically non-significant increase in overall Hb in the iron pot group and a decrease in overall Hb in the aluminum pot group, was likely due to the regression-to-the-mean effect noted by Berti et al. This phenomenon may have been observed in both these studies due to selection and/or analysis of only anemic participants. Both anemic and non-anemic participants likely experienced daily fluctuations in Hb concentrations. However, due to extremely low Hb levels at baseline, fluctuations in Hb were likely more drastic in anemic participants and therefore more likely to be re-measured at a higher level at study endline. As briefly discussed by Berti et al., this regression to the mean effect is often overlooked in research on anemia [36, 45]. An alternative explanation is that statistically significant increases in Hb were only observed among anemic individuals in Arcanjo et al. because these participants had the greatest potential for gain with increased access to bioavailable iron.

More concerning than this regression-to-the-mean effect are the apparent errors in statistical analysis committed in the study by Arcanjo et al. For example, the authors initially state that anemia prevalence decreased from 12.0% (10/93) to 8.4% (7/93) in the iron pot group vs. a reduction from 13.2% (9/68) to 11.8% (8/68) in the aluminum pot group. However, later in the manuscript, they report that anemia prevalence decreased from 12.0% (10/93) to 0.0% (0/93) in the iron pot group vs. a reduction from 13.2% (9/68) to 8.8% (6/68) in the aluminum pot group. In addition, the manuscript reported exclusion of four individuals for refusal to participate and one individual for use of iron supplementation from the iron pot group, but stated that a total of only four participants were excluded from this group (4 +1 = 5) [38]. These errors in statistical analysis call into question the overall integrity of this study.

Also captured by the criterion ‘moderate to high risk of other bias’ was the inappropriate use of per-protocol data analysis. Per-protocol analysis was utilized by Borigato and Martinez [11], Talley et al. [22], Charles et al. 2011 [19], Charles et al. 2015 [37] and Rappaport et al. [24]. Only Adish et al. [12], Sharieff et al. [17] and Geerligs et al. [13] employed intention-to-treat analysis. Devadas et al. [39] and Berti et al. [36] did not specify their approach to data analysis. Intention-to-treat analysis is especially important in studies with high rates of non-compliance and loss-to-follow-up, as the underlying reasons for not adhering to the assigned intervention or control arm may reflect important determinants of intervention success or failure [34].

A final limitation of studies included in this review was limited reporting on iron content and bioavailability of food prepared with iron-containing cookware, ideal cooking conditions, adverse effects and cost-effectiveness. It is possible that most investigators chose to forgo measurement of iron content and bioavailability, because the effect of iron-containing cookware on these parameters has already been relatively well established [14, 18, 29, 46–48]. However, other studies have shown that the iron content and availability of food prepared with iron cookware depends on cooking conditions, including the type of food prepared. Kröger-Ohlsen et al. [49] demonstrated that the amount of iron released by iron cooking pots increases with acidic pH and/or the presence of organic acids (citrate more than lactate). Similarly, Rodriguez-Ramiro et al. [50] found that intestinal absorption of iron released by the Lucky Iron Fish was enhanced 10-fold by ascorbic acid (vitamin C) and inhibited 7.5-fold by tannic acid. Although nine studies in this review reported on the type of food typically prepared with cookware, only the three studies on ingots referenced the addition of citric and/or ascorbic acid.

Regarding adverse effects, Rodriguez-Ramiro et al. [50] found that ascorbic acid reduced the production of harmful reactive oxygen species (ROS) in the intestinal lumen by the Lucky Iron Fish, which were found to be produced at levels similar to standard oral iron supplementation. Besides the generation of ROS by iron cookware, iron overload has long been known as a potential safety concern associated with traditional iron cookware use among the peoples of Sub-Saharan Africa [51–54]. This would be especially important in the setting of a high prevalence of genetic hemoglobinopathies, which can lead to iron overload through increased intestinal iron absorption, inefficient erythropoiesis and recurrent blood transfusion [55]. Individuals with undiagnosed hemochromatosis may also be at increased risk of iron overload with use of iron cookware [56]. Iron overload is of particular concern, as it can cause cirrhosis, liver cancer, heart failure, diabetes mellitus and cancers of other visceral organs [51–55].

In an attempt to capture these potential adverse effects, Geerligs et al. [13] inquired about adverse events with every follow-up survey, but none were reported by study participants. Adish et al. [12] referred all children who became ill to a physician to be evaluated for signs of iron overload. Presumably, no participants suffered from iron overload in this study; however, outcomes regarding adverse effects were not explicitly reported. In Charles et al. [19], three women were deemed outliers and excluded from analyses due to SFe ≥ 130 μg/L. However, all three participants had been randomized to the control group. Therefore, they were not likely suffering from iron overload secondary to ingot use. In Charles et al. [37], assessment of adverse effects was not described as a method employed in the study; however, authors mentioned that no side effects from ingot use were reported by participants nor observed by outcome assessors. These findings are consistent with those of a study by Armstrong et al. [57], in which harmful levels of iron were approached only with simultaneous use of five ingots in water boiled for 60 minutes. However, as noted above, potential conflicts of interest may be associated with studies conducted by Armstrong et al. and the second study conducted by Charles et al. [19, 37, 57]. In addition, only Rappaport et al. measured the prevalence of genetic hemoglobinopathies in study participants; yet, in this study, adverse effects were not assessed [24].

Regarding cost-effectiveness, Adish et al. [12] estimated the cost of distributing one iron pot per household to a population of 10,000 people to be $0.50 per person, given a mean household size of six. Sharieff et al. [17] estimated the cost of iron pots to be $0.6–1.5 per beneficiary per pot, given two to five individuals at risk of IDA per household [18], compared to $1.2 per beneficiary for 12.5 mg of ferrous fumarate per day in a sachet of Sprinkles for two months ([58] in [18]), and $0.27–0.46 per beneficiary for 1.5 iron-folic acid tablets per day for six months ([59] in [17]). Charles et al. [19] and [37] estimated the cost of iron supplementation in Cambodia to be $2–4 per person per month. Based on these data, iron-containing cookware has the potential to be considerably more cost-effective than iron supplementation, especially considering that the lifetime of iron-containing cookware may be several years [12, 19, 37]. However, as Sharieff et al. [17] note, this cost-effectiveness depends on the amount and availability of leached iron, the presence of a sufficient number of individuals at risk of IDA in the household and compliance.

### Implications for research and practice

Future research on the efficacy of iron-containing cookware for the reduction of iron deficiency anemia should focus on minimizing the limitations of research already conducted to date. In particular, more data is needed regarding the effects of age, water quality and genetic hemoglobinopathies. A better description of the factors influencing compliance with use of iron-containing pots is also needed. Indeed, improvements in overall design may improve compliance [16, 18]. Future research should seek to minimize loss-to-follow-up and employ intention-to-treat analysis, in addition to per-protocol analysis, to allow for more valid conclusions. Selection or analysis of only anemic individuals should be performed with caution given the possibility of regression-to-the mean effect in this sub-population. Studies on the adverse effects of food preparation in iron cookware and the relative cost-effectiveness of this intervention would be informative. In addition, greater uniformity of future studies would facilitate more reliable comparisons, such as meta-analysis, especially with regards to the reporting of outcomes. Indeed, the relative change in Hb in the intervention vs. control groups may be a more valuable outcome measure than the mean difference at endline, as the former measure accounts for differences in Hb at baseline, while the latter does not. The study by Sharieff et al. (2008) would be a useful template, as it was the only study in this review found to be of high quality [17].

However, until this research becomes available, public health practitioners should consider the use of iron-containing cookware within the proposed cultural, epidemiological and environmental context. In addition, the benefits must also be carefully weighed against the unknown risks of adverse effects, such as iron overload. Given the potentially significant influence of these contextual factors, the ultimate effect of iron-containing cookware may be modest. Therefore, use of iron-containing cookware may be most effective when combined with other interventions. Perhaps the most notable of these complementary interventions would be food-based strategies to increase the bioavailability of non-heme iron, as addition of ascorbic acid and minimization of tannic acid has obvious benefits for the iron content and bioavailability, and safety, of food prepared with iron-containing cookware.

## Supporting information

S1 Checklist 2009 PRISMA checklist. PRISMA: Preferred Reporting Items for Systematic Reviews and Meta-Analyses.
(DOC)
